# Christison on Poisons

**Published:** 1845-04

**Authors:** 


CHRISTISON ON POISONS.

The first American, from the fourth Edinburgh edition of this unsurpassed work has appeared, in part, in Dr. Bell's Select Medical Library. The part published amounts to 400 pages, and the remainder will follow in a few months, when the American profession will have within their reach the ablest treatise in the English language on the subject of poisons. Y.

